# Well-Being and Associated Factors among Women in the Gender-Segregated Country

**DOI:** 10.3390/ijerph14121573

**Published:** 2017-12-14

**Authors:** Hoda Jradi, Oraynab Abouabbas

**Affiliations:** 1 College of Public Health and Health Informatics, King Saud bin Abdul-Aziz University for Health Sciences, Mail Code 2350, Riyadh 11426, Saudi Arabia; 2 King Abdullah International Medical Research Center, Office of Population Health, Riyadh 11426, Saudi Arabia; Abouabbasor@ngha.med.sa

**Keywords:** women, well-being, mental health, Saudi Arabia, WHO-5 well-being index

## Abstract

Well-being is an essential measure that contributes to the evaluation of the health and quality of life of populations. In 1948, the World Health Organization (WHO) defined health as physical, mental and social well-being. A cross-sectional survey was conducted in Riyadh, Saudi Arabia, between July and September 2015. Women aged 18 years old and above were invited to participate in the study. The data were collected using the WHO’s Well-Being Index questionnaire. Univariate and multivariate logistic regression models were fitted to identify factors that are significantly associated with well-being. A total of 900 women completed the survey. Approximately 58% of the women reported moderate to high (≥50) score of well-being, whereas 41.7% reported ill-being/likely depression. Experiencing violence, living in unfavorable physical conditions and reporting morbidities were shown to be significantly associated with low levels of subjective well-being (ill-being) (*p* < 0.0001). Our study revealed a significant percentage of low levels of well-being among women in Riyadh, Saudi Arabia, and identified the factors associated with them. Further research in this domain is recommended to better investigate additional causes of the low levels of well-being hence help in planning and guiding necessary interventions.

## 1. Introduction

In recent decades, well-being has been increasingly recognized as a major health outcome, and researchers and policy makers have realized the importance of subjective well-being (SWB) as a solid indicator of the economic and social well-being of societies as a whole [1]. SWB is defined as “a broad category of phenomena that includes people’s emotional responses, domain satisfaction and global judgments of life satisfaction” [2].

According to Diener and Suh (1997, p. 191), “subjective well-being research is concerned with individuals’ subjective experiences of their lives. The underlying assumption is that well-being can be defined by people’s conscious experiences—in terms of hedonic feelings or cognitive satisfactions. The field is built on the presumption that to understand the individuals’ experiential quality of well-being, it is appropriate to directly examine how a person feels about life in the context of his or her own standards” [3].

In its constitution in 1948, the World Health Organization (WHO) incorporated the concept of well-being into the general definition of health, defining it as “a state of complete physical, mental and social well-being and not merely the absence of disease or infirmity”; this definition has become the basis for several health-related quality of life measures [4]. The WHO five-item Well-Being Index is one of the tools that assess well-being. This global generic scale, which uses positively phrased questions, was derived from the WHO (Ten) Well-Being Index, which has both negatively and positively phrased questions. The WHO (Ten), in turn, was derived from the WHO’s 28-item Well-Being Questionnaire, which was developed to compare quality of life and well-being among diabetics [5]. 

Although the WHO’s five-item Well-Being Index was originally created as a measure of subjective well-being, several studies have documented its validity in assessing depression, and it has also been used as a screening tool in a variety of populations [6,7,8,9]. 

In Saudi Arabia, health-related quality of life has been well investigated using different tools in different sub-populations [10,11,12]. However, to our knowledge, no studies have been conducted using the WHO-5 Well-Being Index among women; additionally, no studies have investigated the independent effect of material and psychosocial conditions and lifestyle factors on well-being in a nation with a unique culture in which gender roles are enforced via legal and societal measures. Gender segregation and limitations on women’s autonomy may have an impact on quality of life and health outcomes. Hence, it is important to investigate the roles of these factors because of their implications for policy and intervention development in the community and the health system. Moreover, given the conservative nature of the Saudi society and the impact that it may have on women’s well-being, this study aimed at assessing the well-being of women in Riyadh [6] and identifying associated risk factors using the validated Arabic version of the WHO-5 Well-Being Index [8], thus filling a gap in the literature on women’s health. 

## 2. Methods

Well-being and associated factors were investigated in a Saudi population sample of women aged 18–70 years residing in the city of Riyadh, Saudi Arabia. The data were collected from July to September 2015 using an interviewer-administered questionnaire. The questionnaire was in the Arabic language, and all questions were checked for the clarity of the language used and word choice by testing it on a group of 30 women. Modifications were made to the questionnaire as suggested. The reliability of the instrument was checked by administering the questionnaire twice to the same group of women (*n* = 25) and within a one-week interval between the two interviews. The sampling included stratification of the city into five regions (northern, eastern, western, central, and southern), random selection of two large shopping centers in each region, random selection of days of the week and hours of the day for data collection, and random selection of women entering the selected shopping facilities. Shopping malls are a major destination for women of all ages and all socioeconomic backgrounds in the city of Riyadh. A total of 950 women were asked to participate in the study, and 900 gave consent to be interviewed, for an overall response rate of 95%. Well-being was assessed by administering the Arabic version of the 5-item World Health Organization (WHO-5) Well-Being Index. This index has been validated, translated into many languages, and used in studies in different countries [13]. Responses to the five questions are recoded from 0 (no, never) to 5 (always), with a possible range of scores between 0 and 25. The scores were converted into percentages and the obtained percentage scores were reported in terms of two categories: a moderate to high level of well-being (≥50) and ill-being/likely depression (<50).

All procedures performed in studies were in accordance with the ethical standards of the institutional research committee and with the 1964 Helsinki declaration and its later amendments or comparable ethical standards. Informed consent was obtained from all participants. The Study was approved by the Institutional Review Board at King Abdullah International Medical Research Center (SP 14/023/R).

### 2.1. Socioeconomic Status

The age of the participants was grouped into four categories: 18–28, 29–39, 40–49, and ≥50 years old. Educational level was categorized into five classes: illiterate, elementary school, middle school, high school, and college or higher. Employment status was derived from a survey question regarding whether the participant was employed, unemployed, a student, or retired. The participants’ body mass index (BMI) was calculated from self-reported weight and height as kg/m^2^. The participants were classified according to WHO recommendations into three categories: underweight (<18.5 kg/m^2^), normal (<25 kg/m^2^), overweight (25–29.9 kg/m^2^) and obese (≥30 kg/m^2^).

### 2.2. Physical Environment

The physical environment was described by asking the participants “Do you have disturbance in or around your place of residence or work from the following sources?”. The listed sources were noise or exhaust from surrounding streets or nearby factories, smelly fumes or gases, or other disturbances, with the options of “Yes” or “No”. The responses were then summarized to reflect a good or poor physical environment. 

### 2.3. Adverse Experiences and Health Status

Adverse experiences were assessed by two main questions. First, the respondents were asked whether they had experienced an act of violence in the past twelve months. Women who responded “Yes” to the question were asked to specify the type of violence that they experienced. The second question about an adverse experience was “Have you ever experienced a physical injury?”. The reported physical injuries were classified by reported cause. Health status was assessed by the following question: “Do you currently have a disease/health condition that requires treatment?”. The response choices were “Yes” or “No”. 

### 2.4. Statistical Analysis

Analyses were performed using the STATA statistical software (version 13; College Station, TX, USA). Descriptive statistics as the means and frequencies were calculated for all study variables where applicable. The differences in the categories of reported well-being across study variables (demographic characteristics, socioeconomic status, physical environment, adverse experiences, and health status) were assessed. The chi-square test for categorical variables was used to examine significant differences in the parameters. 

Univariate and multivariate logistic regressions analyses were performed with reported well-being as the dependent variable to examine the simultaneous effects of the studied factors on the likelihood of moderate/high well-being vs. ill-being/likely depression. The odds ratios and 95% confidence intervals of a moderate/high level of well-being compared to ill-being/likely depression were calculated for a range of study variables. First, all demographic variables that showed significance in the univariate analysis were entered into the logistic model. Next, the variables related to the physical environment were introduced, followed by adverse experiences and reported morbidity status. Stepwise backward elimination of non-significant variables was applied (significance level set at *p* < 0.05). A total of 900 participants with full data for all variables were considered. 

## 3. Results

Table 1 presents the number of participants in the different age groups (mean age was 29.3 years; SD ± 9.7) and the frequencies and distributions of the characteristics for the total sample. The majority of the women were high school graduates (46.6%), single (54.4%), and employed (44.7%). The mean BMI was 24.5 kg/m^2^. Approximately 43.7% said they lived or worked in an unfavorable physical environment. Forty-six percent of the surveyed women reported having one or more morbidity, and 16.5% reported experiencing violence (physical or emotional/psychological) in the previous 12 months. The overall prevalence of ill-being/likely depression among this sample of women was 41.7%.

Table 2 displays the classification of the participants by well-being status. The proportion of moderate to high well-being did not vary significantly by age, marital status, employment status, income, or level of education. The proportion of women with ill-being/likely depression was significantly higher among those who were overweight/obese (*p* = 0.033), worked or lived in an unfavorable environment (*p* = 0.002), reported at least one morbidity (*p* = 0.004), and reported experiencing physical or emotional/psychological violence (*p* = 0.002). 

Table 3 shows the unadjusted and adjusted odds ratios of reporting ill-being/likely depression. The unadjusted odds ratios were the highest for the participants who reported experiencing physical violence (OR = 2.1; 95% CI: 1.271–3.573), followed by those who reported experiencing emotional/psychological violence (OR = 1.8; 95% CI: 1.300–2.638). The odds of reporting ill-being among those with a high BMI were 1.4 times the odds among those with a normal BMI (95% CI: 1.092–1.862). Living or working in an unfavorable physical environment significantly (1.5-fold) increased the risk of ill-being (95% CI: 1.178–2.013). In addition, the risk of ill-being was 1.5-fold (95% CI: 1.187–2.325) higher among those reporting a morbidity compared to those with no morbidity.

In the adjusted final model, experiencing violence, living in an unfavorable physical environment, and having morbidity were significantly associated with ill-being/likely depression. Adjusting for all other factors, the risk of ill-being/likely depression did not reach significance for those with a BMI that was higher than normal. The final model showed a good fit (Hosmer–Lemeshow Goodness-of-Fit test; *p* > 0.05).

## 4. Discussion

The WHO-5 Well-Being Index has been used in research worldwide, not only as a generic tool for assessing the well-being of various population groups but also as a depression screening tool as well as an outcome measure in several clinical trials [13]. Examples of the study fields in which the WHO-5 item questionnaire has been applied include cardiology [14], oncology [15], psychology, geriatrics [8], diabetes [16], and occupational health [17]. 

In this study, we measured the general well-being among women in Riyadh, and our results showed that 58.3% of women reported a moderate to high (≥50) score of well-being and 41.7% reported ill-being/likely depression. 

Violence against women has been shown to have profound emotional and psychological consequences for women’s mental health, including an increased risk of depression, post-traumatic stress disorder, anxiety, eating and sleep disorders, lowered self-esteem, suicidal ideation and behavior and alcohol and drug misuse [18,19,20,21,22,23]. Similarly, our findings showed that experiencing violence was a significant predictor of well-being status. Women who had experienced violence were more likely to report ill-being compared to women who had not. Unfortunately, in the Middle East domestic violence tends to be more aggressive and acceptable than in other regions [24], yet silence and secrecy still often surround cases of violence. This can be attributed to the patriarchal nature of Arab societies, including Saudi society, where stigma and shame are associated with violence. A vast body of research has documented the immense effect that violence has on women’s health and well-being; however, it is still widely neglected and under-researched. This shows the importance of highlighting this issue, taking the necessary actions and planning effective interventions. 

As for unfavorable physical environment, our findings showed it to be significantly associated with women’s ill-being, which is aligned with the literature on this issue [25,26,27]. Women living/working in an unfavorable environment were 1.4 times more likely to report ill-being/depression compared to women living in a favorable physical environment. According to Wilkinson and colleagues (2003), neighborhoods with poor housing quality, limited or restricted access to quality health services, limited or no public transportation systems, and a lack of services such as recreation facilities, libraries, and green spaces are considered negative determinants of health [28]. 

In their study, Maas and colleagues (2006) found a positive correlation between the extent of green areas in living environments and people’s perceived general health [29]. Mental health problems were found to be less prevalent in urban places that have gardens and open spaces [30]. Moreover, a systematic review of the benefits of exposure to the natural environment for health and well-being showed a positive influence of exposure to the natural environment on people’s psychological well-being [25]. In this context, it has also been argued that natural settings or natural environments promote physical activity and exercise, which ultimately improve health and overall well-being [30,31]. Obesity rates can also diminish with improvement in physical activity in a gender-segregated country where physical education for women is prohibited based on religious grounds [32]. Overweight and obesity have been linked with poor mental health due to their implication on an individual’s quality of life [33]. Accordingly, this should guide decision and policy makers and help them better plan and establish services that can address the needs of the population in Saudi Arabia. 

Morbidity was also found to be a significant predictor of women’s ill-being in Riyadh. Evidence on the effect of morbidity on mental health and ratings of health in general has been well established [34,35,36,37,38]. Studies conducted among the general population reported that chronic conditions can have a negative impact on health perceptions [35,36] and quality of life [34]. Another study, among a community sample in Hong Kong, showed a higher prevalence of depressive symptoms among people with one or more chronic physical illnesses; depressive symptoms were more prevalent among females [33]. Depression was also found to be common in a sample of women with breast cancer attending an outpatient clinic in Jeddah, Saudi Arabia [33]. The poor well-being perceived by this group of women may reflect a disease burden that warrants further attention and investigation. Women in Saudi Arabia may find healthcare services difficult to access due to imposed gender segregation norms and male guardianship. 

One limitation of this study can be linked to its self-reported nature, which makes participants more prone to information and recall bias. However, it should be noted that the survey underwent a validity and reliability check in this population. Also, drawing the sample from shopping centers can be considered a limitation of the study. Although it is reassuring that the sample is demographically similar to the actual demographic characteristics of Saudi Arabia in terms of education, employment and marital status [39,40]. Another limitation is the cross-sectional nature of the study design, which does not allow conclusions to be drawn on causality or the temporality between the dependent and independent variables; however, it revealed low levels of well-being among women in Riyadh and identified the factors associated with ill-being. National policy reforms and programs that can support and empower women are greatly needed. Policy and decision makers should be well informed about the current situation; they should also be made aware of the serious impact of the built environment on mental health to help them better plan and invest in services that can promote mental well-being not only for women but also for the general population as a whole. 

## 5. Conclusions

Our study revealed a significant percentage of low levels of well-being among women in Riyadh, Saudi Arabia, and identified the factors associated with them. Further research in this domain is recommended to better investigate additional causes of the low levels of well-being and hence help in planning and guiding necessary interventions in a unique environment where gender roles and expectations are embedded in the legal and societal system. This should guide decision and policy makers and help them better plan and establish services that can address the needs of the population in Saudi Arabia.

## Figures and Tables

**Table 1:** Characteristics of study participants (N = 900).

Characteristic	N (%)
**Age (years)**	
18–28	385 (42.8)
29–39	233 (25.9)
40–49	185 (20.6)
≥50	97 (10.8)
**Marital Status**	
Single	481 (53.4)
Married	333 (37.0)
Separated/Divorced/Widowed	86 (9.6)
**Educational Level**	
Illiterate	15 (1.7)
Elementary	66 (7.3)
Middle School	50 (5.6)
High School	419 (46.6)
College or more	350 (38.9)
**Employment Status**	
Employed	402 (44.7)
Not Employed	459 (51.0)
Student	39 (4.3)
Retired	2 (0.2)
**Body Mass Index (N = 848)**	
Normal Weight	431 (50.8)
Overweight/Obese	417 (49.2)
**Physical Environment**	
Favorable	507 (56.3)
Unfavorable	393 (43.7)
**Reported Morbidities**	
No	486 (54.0)
Yes	414 (46.0)
**Experienced Violence**	
No	751 (83.4)
Yes	149 (16.5%)
**Well-Being**	
Moderate to High (≥50)	525 (58.3)
Ill-Being/Likely Depression (<50)	375 (41.7)

**Table 2:** Comparison of socioeconomic status, physical environment, lifestyle factors, and health status between women with good and poor self-rated heath (N = 900).

Variables	Well-Being		
	Moderate to High	Ill-Being/Likely Depression	*p*-Value
**Age (years) (µ = 29.3; SD = 9.7)**			0.432
18–28	300	202	
29–39	141	119	
40–49	59	36	
≥50	25	18	
**Marital Status**			0.514
Single	288	193	
Married	186	147	
Separated/Divorced/Widowed	51	35	
**Educational Level**			0.224
Illiterate	6	9	
Elementary	33	33	
Middle School	26	24	
High School	254	165	
College or more	206	144	
**Employment Status**			0.281
Employed	275	184	
Not Employed	229	171	
Student	21	18	
Retired	0	2	
**Body Mass Index (N = 848)**			0.033
Normal Weight	249	182	
Overweight/Obese	131	148	
Obese	62	76	
**Physical Environment**			0.002
Favorable	319	188	
Unfavorable	187	206	
**Reported Morbidities**			0.004
No	310	176	
Yes	198	216	
**Experienced Violence**			0.002
No	457	94	
Yes	68	81	

**Table 3:** Adjusted and un-adjusted Odds Ratios (OR) for “ill-being/likely depression” compared to “moderate to high” well-being among Saudi women (N = 900).

	Well-Being			
	U-OR (95% CI)	*p*-Value	A-OR (95% CI)	*p*-Value
**Body Mass Index**		0.009		0.10
Normal Weight	Ref.		Ref.	
Overweight/Obese	1.4 (1.092–1.862)		1.2 (0.981–1.090)	
**Experienced Violence**		0.02		0.011
No	Ref.		Ref.	
Yes	1.8 (1.300–2.638)		1.6 (1.116–2.317)	
**Physical Environment**		0.002		0.011
Favorable	Ref.		Ref.	
Unfavorable	1.5 (1.178–2.013)		1.4 (1.084–1.872)	
**Reported Morbidities**		0.004		0.012
No	Ref.		Ref.	
Yes	1.5 (1.187–2.325)		1.5 (1.097–2.168)	

## References

[1] Hayo B., Seifert W. (2003). Subjective economic well-being in Eastern Europe. J. Econ. Psychol..

[2] Diener E., Suh E., Lucas R., Smith H. (1999). Subjective Well-Being: Three Decades of Progress. Psychol. Bull..

[3] Diener E., Suh E. (1997). Measuring quality of life: Economic, social, and subjective indicators. Soc. Indic. Res..

[4] World Health Organization (2006). Constitution of the World Health Organization. http://www.who.int/governance/eb/who_constitution_en.pdf.

[5] Mavaddat N., Valderas J.M., van der Linde R., Khaw K.T., Kinmonth A.L. (2014). Association of self-rated health with multimorbidity, chronic disease and psychosocial factors in a large middle-aged and older cohort from general practice: A cross-sectional study. BMC Fam. Pract..

[6] Krieger T., Zimmermann J., Huffziger S., Ubl B., Diener C., Kuehner C., Grosse Holtforth M. (2014). Measuring depression with a well-being index: Further evidence for the validity of the WHO Well-Being Index (WHO-5) as a measure of the severity of depression. J. Affect. Disord..

[7] Allgaier A.K., Pietsch K., Fruhe B., Prast E., Sigl-Glockner J., Schulte-Korne G. (2012). Depression in pediatric care: Is the WHO-Five Well-Being Index a valid screening instrument for children and adolescents?. Gen. Hosp. Psychiatry.

[8] Sibai A.M., Chaaya M., Tohme R.A., Mahfoud Z., Al-Amin H. (2009). Validation of the Arabic version of the 5-item WHO Well Being Index in elderly population. Int. J. Geriatr. Psychiatry.

[9] Henkel V., Mergl R., Kohnen R., Allgaier A.K., Moller H.J., Hegerl U. (2004). Use of brief depression screening tools in primary care: Consideration of heterogeneity in performance in different patient groups. Gen. Hosp. Psychiatry.

[10] Ahmed A.E., Alharbi A.G., Alsadhan M.A., Almuzaini A.S., Almuzaini H.S., Ali Y.Z., Jazieh A.R. (2017). The predictors of poor quality of life in a sample of Saudi women with breast cancer. Breast Cancer.

[11] AlBuhairan F., Nasim M., Al Otaibi A., Shaheen N.A., Al Jaser S., Al Alwan I. (2016). Health related quality of life and family impact of type 1 diabetes among adolescents in Saudi Arabia. Diabetes Res. Clin. Pract..

[12] Abolfotouh M.A., Al-Khowailed M.S., Suliman W.E., Al-Turaif D.A., Al-Bluwi E., Al-Kahtani H.S. (2012). Quality of life in patients with skin diseases in central Saudi Arabia. Int. J. Gen. Med..

[13] Topp C.W., Østergaard S.D., Søndergaard S., Bech P. (2015). The WHO-5 Well-Being Index: A Systematic Review of the Literature. Psychother. Psychosom..

[14] Birket-Smith M., Hansen B.H., Hanash J.A., Hansen J.F., Rasmussen A. (2009). Mental disorders and general well-being in cardiology outpatients—6-year survival. J. Psychosom. Res..

[15] Hoffman C.J., Ersser S.J., Hopkinson J.B., Nicholls P.G., Harrington J.E., Thomas P.W. (2012). Effectiveness of mindfulness-based stress reduction in mood, breast-and endocrine-related quality of life, and well-being in stage 0 to III breast cancer: A randomized, controlled trial. J. Clin. Oncol..

[16] Logtenberg S.J., Kleefstra N., Houweling S.T., Groenier K.H., Gans R.O., Bilo H.J. (2010). Health-related quality of life, treatment satisfaction, and costs associated with intraperitoneal versus subcutaneous insulin administration in type 1 diabetes. Diabetes Care.

[17] Feicht T., Wittmann M., Jose G., Mock A., von Hirschhausen E., Esch T. (2013). Evaluation of a seven-week web-based happiness training to improve psychological well-being, reduce stress, and enhance mindfulness and flourishing: A randomized controlled occupational health study. Evid.-Based Complement. Altern. Med..

[18] Al Dosary A.H. (2016). Health Impact of Domestic Violence against Saudi Women: Cross Sectional Study. Int. J. Health Sci..

[19] Kumar A., Haque Nizamie S., Srivastava N.K. (2013). Violence against women and mental health. Ment. Health Prev..

[20] World Health Organization (2013). Global and Regional Estimates of Violence against Women: Prevalence and Health Effects of Intimate Partner Violence and Non-Partner Sexual Violence.

[21] Haddad L.G., Shotar A., Younger J.B., Alzyoud S., Bouhaidar C.M. (2011). Screening for domestic violence in Jordan: Validation of an Arabic version of a domestic violence against women questionnaire. Int. J. Womens Health.

[22] Douki S., Nacef F., Belhadj A., Bouasker A., Ghachem R. (2003). Violence against women in Arab and Islamic countries. Arch. Women’s Ment. Health.

[23] Haj-Yahia M.M. (2000). Implications of Wife Abuse and Battering for Self-Esteem, Depression, and Anxiety as Revealed by the Second Palestinian National Survey on Violence against Women. J. Fam. Issues.

[24] Lafta R.K. (2008). Intimate-partner violence and women’s health. Lancet.

[25] Bowler D.E., Buyung-Ali L.M., Knight T.M., Pullin A.S. (2010). A systematic review of evidence for the added benefits to health of exposure to natural environments. BMC Public Health.

[26] Mitchell R., Popham F. (2007). Greenspace, urbanity and health: Relationships in England. J. Epidemiol. Community Health.

[27] Evans G.W. (2003). The built environment and mental health. J. Urban Health Bull. N. Y. Acad. Med..

[28] Wilkinson R.G., Marmot M. (2003). Social Determinants of Health: The Solid Facts.

[29] Maas J., Verheij R.A., Groenewegen P.P., De Vries S., Spreeuwenberg P. (2006). Green space, urbanity, and health: How strong is the relation?. J. Epidemiol. Community Health.

[30] Pretty J., Peacock J., Hine R., Sellens M., South N., Griffin M. (2007). Green exercise in the UK countryside: Effects on health and psychological well-being, and implications for policy and planning. J. Environ. Plan. Manag..

[31] Kaczynski A.T., Henderson K.A. (2007). Environmental correlates of physical activity: A review of evidence about parks and recreation. Leis. Sci..

[32] AlQuaiz A.M., Siddiqui A.R., Qureshi R.H., Fouda M.A., AlMuneef M.A., Habib F.A., Iqbal M. (2014). Turkistani. Women Health in Saudi Arabia: A review of Non-communicable Diseases and Their Risk Factors. Pak. J. Med. Sci..

[33] Scott K.M., Bruffaerts R., Simon G.E., Alonso J., Angermeyer M., de Girolamo G., Demyttenaere K., Gasquet I., Haro J.M., Karam E. (2007). Obesity and mental disorders in the general population: Results from the world mental health surveys. Int. J. Obes..

[34] Nan H., Lee P.H., McDowell I., Ni M.Y., Stewart S.M., Lam T.H. (2012). Depressive symptoms in people with chronic physical conditions: Prevalence and risk factors in a Hong Kong community sample. BMC Psychiatry.

[35] Al-Zaben F., Sehlo M., El-deek B., Koenig H. (2015). Depressive symptoms, correlates, and the marital relation-ship in women with breast cancer in Saudi Arabia. Women Health Open J..

[36] Sprangers M.A., de Regt E.B., Andries F., van Agt H.M., Bijl R.V., de Boer J.B., Foets M., Hoeymans N., Jacobs A.E., Kempen G.I. (2000). Which chronic conditions are associated with better or poorer quality of life?. J. Clin. Epidemiol..

[37] Lyons R.A., Lo S.V., Littlepage B. (1994). Comparative health status of patients with 11 common illnesses in Wales. J. Epidemiol. Community Health.

[38] Stewart A.L., Greenfield S., Hays R.D., Wells K., Rogers W.H., Berry S.D., McGlynn E.A., Ware J.E. (1989). Functional status and well-being of patients with chronic conditions: Results from the Medical Outcomes Study. JAMA.

[39] World Bank Group (2016). Getting to Equal: Women, Business, and the Law.

[40] Demography Survey 2016; General Authority for Statistics: Saudi Arabia. https://www.stats.gov.sa/en.

